# Evaporation of As and Sn from Liquid Iron: Experiments and a Kinetic Model during Top-Blown Oxygen Steelmaking Process

**DOI:** 10.3390/ma15144771

**Published:** 2022-07-07

**Authors:** Dong-Hyun Kim, Won-Bum Park, Sang-Chae Park, Youn-Bae Kang

**Affiliations:** 1 Graduate Institute of Ferrous & Energy Materials Technology, Pohang University of Science and Technology, Pohang 37673, Gyeongbuk, Korea; kdh0516@postech.ac.kr (D.-H.K.); wbpark93@postech.ac.kr (W.-B.P.); 2 Technical Research Laboratories, POSCO, Pohang 37859, Gyeongbuk, Korea; sangchae79@posco.com

**Keywords:** tramp elements, As, Sn, evaporation, top-blown oxygen steelmaking, impact zone, droplet generation

## Abstract

Evaporation kinetics of tramp elements (M = As and Sn) in liquid iron were investigated by high-temperature gas–liquid reaction experiments and a phenomenological kinetic model. Residual content of As or Sn in the liquid iron ([pct M]) during the evaporation was measured in the temperature range of 1680 ${}^{{}^{\circ}}$C to 1760 ${}^{{}^{\circ}}$C. [pct As] and [pct Sn] decreased faster as the reaction temperature and [pct C]${}_{0}$ increased. Assuming first-order reaction kinetics, the apparent rate constants ($k_{M}$) were obtained at each reaction temperature and [pct C]${}_{0}$. [pct M] in a liquid iron during the top-blown oxygen steelmaking process was simulated, with an emphasis on enlarging the reaction surface area by forming a large number of liquid iron droplets. The surface area and the droplet generation rate were obtained based on the oxygen-blowing condition. The whole surface area increased up to ∼163 times the initial liquid iron (bath) surface area, due to the generation of the droplets. Using the $k_{M}$ obtained in the present study, the evaporation of M during the top-blown oxygen steelmaking process for 200 tonnes of liquid iron was simulated. For a condition of [pct M]${}_{0}$ = 0.005 (M = As and Sn), As and Sn could be removed from the liquid iron, which was seen to be much improved by the consideration of the droplet generation. However, additional actions are required to improve the evaporation rate, as the evaporation rate in the BOF process was not fast enough to be practically considered.

## 1. Introduction

Due to the depletion of high-quality iron ores with a low level of impurities, the use of low-quality ores with impurities is required. The impurities become natural tramp elements in the liquid iron/steel, unless those are removed before casting the steel. Among those impurities, As and Sn are those found in the iron ore available in western Australia [1]. It would be the best scenario if the tramp elements could be removed in the ore blending/sintering process. Otherwise, it is necessary to find a feasible way to remove them before they enter the liquid iron.

It was previously reported that the conventional molten lime flux-based refining processes might not be feasible due to the too low distribution ratio of the tramp elements between liquid iron and the molten fluxes [2,3,4]. For example, a recent study by the present authors showed that the distribution ratio of Sn between a liquid iron and CaO–CaF${}_{2}$ flux ($L_{\mathrm{Sn}}={\left(\mathrm{pct}\;\mathrm{Sn}\right)}_{\mathrm{flux}}/{\left[\mathrm{pct}\;\mathrm{Sn}\right]}_{\mathrm{iron}}$) was in the order of 10${}^{-3}$ to 10${}^{-4}$ in the temperature range of 1400∼1600 ${}^{{}^{\circ}}$C [4]. This was attributed to the very low solubility of Sn in the molten flux [2,3,4] and the noble character of Sn compared to that of Fe. Similar behavior was also reported for As [2]. Moreover, the distribution ratio was generally decreased as the oxygen potential in the system increased, since those elements dissolve in the molten flux as anions below the critical oxygen potential [2,4]. During the investigation of the distribution of Sn by the present authors [4], a non-negligible portion of Sn disappeared from the whole system (the liquid iron and the molten flux), although those were hardly found in the molten flux. Similar phenomena were found for As and Pb [5]. It was likely that those elements evaporated.

Evaporation kinetics and mechanism of Sn and Cu from liquid iron were investigated in the present authors’ laboratory [6,7,8,9,10,11]. It was found that increasing reaction temperature and [pct C] in the iron increased the evaporation rate, which was also reported by other researchers [12,13,14]. Coupled roles of C and S on the evaporation of Sn and Cu were elucidated for the first time [6,7,8,9,10]. In these studies, the evaporation tests were carried out using a laboratory-scale electromagnetic levitation technique, which inherently employed a small-sized metal droplet with a high surface-to-volume ratio. A relatively faster reaction rate compared to other investigations [15,16] was achieved. It was presumably due to the large surface-to-volume ratio in the present authors’ investigations [6,7,8,9,10]. Moreover, intensive internal circulation by the electromagnetically induced melting resulted in the elimination of liquid phase mass transport resistance [6,17]. In the absence of the electromagnetic force, Marangoni flow may affect the liquid phase mass transport [18]. This means that the evaporation rate of the tramp elements in liquid iron is likely to be faster if a large number of droplets are generated. Moreover, the evaporation rate becomes faster at high temperature and high C content in the liquid iron [8,9,11]. Therefore, the evaporation of tramp elements may be favorable during the Basic Oxygen Furnace (BOF) steelmaking process. In the BOF process, a large amount of iron droplets is generated by oxygen top-blowing [19,20]. The estimation of the mass or volume of the droplets during the oxygen top-blown process is available (described in detail in Section 5). In particular, increasing the surface area of the liquid iron with a large number of droplets seems to be advantageous for accelerating evaporation.

In the present study, the evaporation of tramp elements (As and Sn) from liquid iron in the BOF process was investigated. First, the evaporation rate of these elements was measured experimentally, and the apparent rate constants were obtained. Second, mass, temperature, and surface area of ejected iron droplets during a top-blown oxygen steelmaking process were estimated by utilizing models available in the literature [19,20,21,22,23]. Those two were coupled to simulate the evaporation rate of the tramp elements during the BOF process. It was assessed how much these tramp elements could be removed during the top-blowing BOF process via the droplets. Due to the evaporation of heavy metal, some environmental issues may arise. This must be controlled by installing proper dust collecting facilities such as an electrostatic precipitator [24], etc., if the proposed evaporation refining of tramp elements are feasible.

## 2. Materials and Methods

### 2.1. Sample Preparation

Several Fe–C–As–Sn alloys were prepared in an induction melting furnace under a purified Ar atmosphere. C content in the alloys was controlled either by adding electrolytic iron (99.9 pct, Blyth & Co., Ltd., Yokohama, Japan) and some graphite powder (99.97 pct, Bansukcarbon, Korea) in a magnesia crucible (OD 60 mm × ID 50 mm × H 100 mm) or by melting the electrolytic iron in a graphite crucible (OD 60 mm × ID 45 mm × H 100 mm). Certain amounts of As (99.999 pct, RND Korea Corp., Gwangmyeong-si, Korea) or Sn (99.99 pct, RND Korea Corp., Korea) were added to the melt. The melt was held for 20 to 30 min at each temperature (see Table 1 for the holding temperature and the initial composition). Subsequently, small portions of the melt were sampled by quartz tubes to obtain bars of 4 × 10${}^{-3}$ m diameter. Those were cut and ground into smaller pieces of (6 ± 0.5) × 10${}^{-4}$ kg to be suitable for subsequent electromagnetic levitation.

### 2.2. Experimental Apparatus and Procedure

An electromagnetic levitation technique was used based on an RF generator (30 kW, 260 kHz, PSTEK, Gunpo-si, Korea). A piece of the prepared sample was located at a position of levitation by a quartz-made cup, and then the power of the RF generator was turned on. During the levitation and subsequent melting, Ar gas was supplied to the reaction chamber at a rate of 1.0 L min${}^{-1}$ using a capillary flowmeter. The Ar gas was purified by passing through the CaSO${}_{4}$ column, and Mg chips heated at 500 ${}^{{}^{\circ}}$C to remove traces of moisture and oxygen. The temperature of the levitated droplet was measured by a two-color pyrometer (Raytek, RAY2CBG, Santa Cruz, CA, USA), and was controlled by adjusting the RH generator power manually. The pyrometer was calibrated by measuring the melting temperature of pure Ti, which was independently measured by a B-type thermocouple. Uncertainty in the temperature was estimated to be ±10 ${}^{{}^{\circ}}$C. In the present study, all experiments were carried out in the range of 1680∼1760 ${}^{{}^{\circ}}$C. After a predetermined time passed up to 600 s, the RF generator was turned off and the droplet was quenched into a water-cooled copper mold. The sample was collected, and As and Sn contents in the droplet were analyzed by ICP-AES (Thermo-Fisher Scientific ICAP 6500, Waltham, MA, USA). C content was analyzed using the C/S combustion method (LECO CS-844, St-Joseph, MI, USA).

## 3. A Preliminary Test

**Figure 1:**
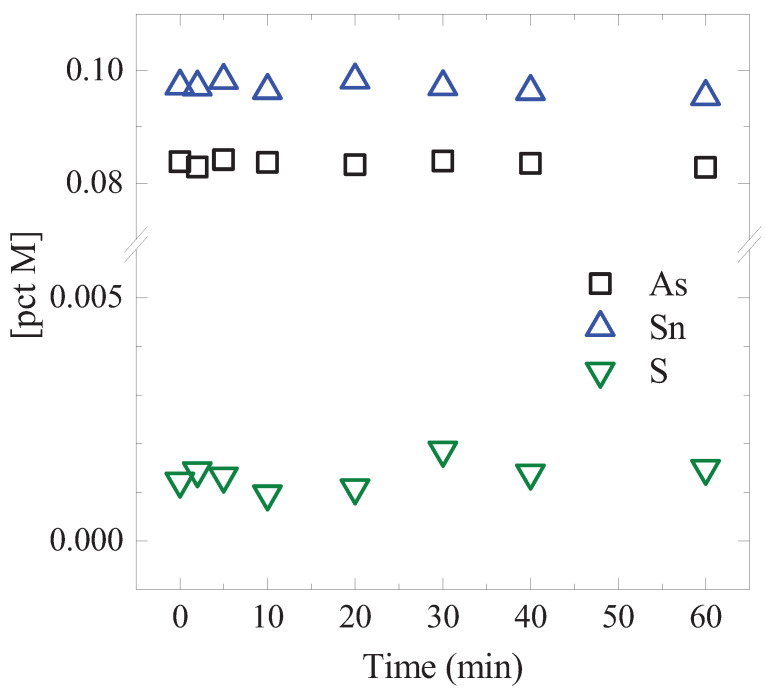
Results of the preliminary experiment on the evaporation of As and Sn in a C-saturated liquid iron at 1400 ${}^{{}^{\circ}}$C. (color online).

Evaporation of Sn in low-S containing liquid iron was slow due to the low vapor pressure of Sn [6,7,8,12,15]. In order to qualitatively assess the rate of evaporation of As and Sn, a simple preliminary test was carried out. A liquid iron that contained As and Sn was melted in a graphite crucible for an hour at 1400 ${}^{{}^{\circ}}$C. Mechanical stirring was executed by a graphite impeller in order to accelerate the mass transport of the elements in the iron. The liquid iron was periodically sampled and its composition was analyzed (analysis methods are described in Section 2.2). The result is shown in Figure 1. [pct As] and [pct Sn] did not change noticeably. S was not added to the liquid iron, but its content was analyzed for a purpose. This was because S can affect the evaporation rate of Sn, as was reported previously [6,7,8,12,15]. In this preliminary test, [pct S] did not change noticeably in the range of 0.001 to 0.002. Since [pct S] was low, the evaporation of Sn by forming SnS(g) was unlikely [7,11]. It is not known whether S would accelerate the evaporation of As, to the best knowledge of the present authors. It can be concluded that the evaporations of As and Sn were slow, and their overall evaporations should be accelerated by other means, for example, increasing the reaction area by forming a large number of droplets in the BOF.

## 4. Evaporation of As and Sn from a droplet

The evaporation rate of As and Sn from a liquid iron droplet with different C content was measured using an electromagnetic levitation melting technique (see Section 2.2). Experimental details are similar to those employed in previous studies by Jung et al. [6,7,8].

### 4.1. Effect of C Content on As and Sn Evaporations

**Figure 2:**
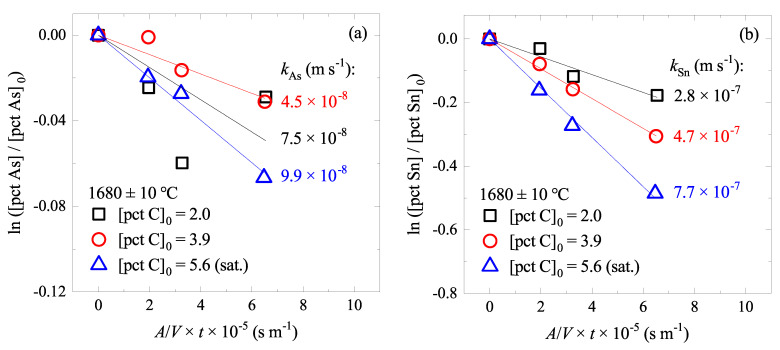
Changes of (**a**) [pct As]/[pct As]${}_{0}$ and (**b**) [pct Sn]/[pct Sn]${}_{0}$ in Fe–C–As–Sn alloys with various [pct C]${}_{0}$ at 1680 ± 10 ${}^{{}^{\circ}}$C. (color online).

Figure 2 shows the changes of As and Sn contents ([pct As] and [pct Sn]) in the liquid alloys with different initial C contents ([pct C]${}_{0}$) at 1680 ${}^{{}^{\circ}}$C. It was confirmed that increasing [pct C]${}_{0}$ increased the evaporation rate of As and Sn, except for the As evaporation at [pct C]${}_{0}$ = 2.0. The following two reactions were assumed:

(1) $$\underline{\mathrm{As}}=\mathrm{As}(g)$$

(2) $$\underline{\mathrm{Sn}}=\mathrm{Sn}(g)$$

It is thought that as [pct C]${}_{0}$ increased, the repulsive force between C and As and between C and Sn in droplets became stronger and their evaporation became more active [25]. This was similar to the previous cases of Cu and Sn evaporation [7,9]. Therefore, As and Sn evaporations are expected to be favorable at a high C content in liquid iron. In view of the oxygen-blown BOF, the early stage of the steelmaking would be favorable where [pct C] is high, if the temperature is not considered. However, as will be shown in the next section, the evaporation should be faster at a higher temperature.

Assuming the evaporation of M (= As or Sn) is a first-order reaction, the rate may be simply described as:

(3) $$\frac{d[\mathrm{pct}\;M]}{dt}=-\frac{A}{V}\times k_{M}\times \left[\mathrm{pct}\;M\right]$$

where *A*, *V*, $k_{M}$, and *t* are the reaction interfacial area (m${}^{2}$), the volume of a droplet (m${}^{3}$), the apparent rate constant of the evaporation reaction (m s${}^{-1}$), and the reaction time (s), respectively. [pct M] can be expressed by the following equation:

(4) $$\ln\frac{[\mathrm{pct}\;M]}{[\mathrm{pct}\;{M]}_{0}}=-\frac{A}{V}\times k_{M}\times t$$

The droplet mass was assumed to be 6 × 10${}^{-4}$ kg, and the droplet density difference due to the different C content was taken into account [26]. $k_{M}$ for each case was then obtained from the slope of the plot shown in Figure 2. It was found that $k_{\mathrm{As}}=4.5-9.9\times 10^{-8}$ m s${}^{-1}$ and $k_{\mathrm{Sn}}=2.8-7.7\times 10^{-7}$ m s${}^{-1}$. The latter is similar to that reported by Jung et al. at *T* = 1600 ${}^{{}^{\circ}}$C and [pct C]${}_{0}$ = 0 ($3.5\times 10^{-7}$ m s${}^{-1}$) [7].

### 4.2. Effect of Temperature on As and Sn Evaporations

**Figure 3:**
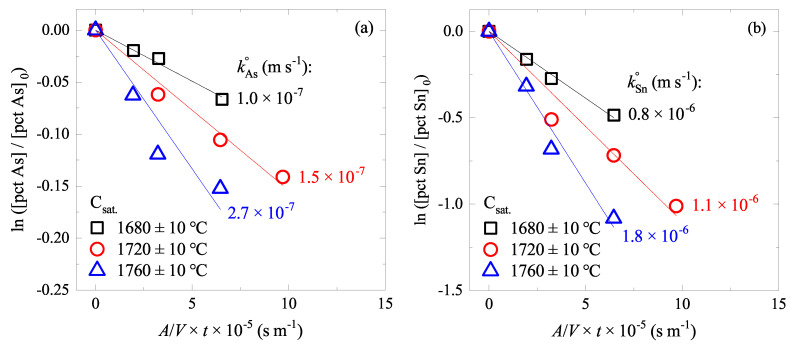
Changes of (**a**) [pct As]/[pct As]${}_{0}$ and (**b**) [pct Sn]/[pct Sn]${}_{0}$ in C-saturated Fe–C–As–Sn alloys at various temperatures. (color online).

In a similar manner, the changes of As and Sn contents in liquid Fe–C–As–Sn alloys of C saturation at various temperatures were measured and the data are shown in Figure 3. Increasing temperature increased the evaporation rate of As and Sn: $k_{\mathrm{As}}=1.0$–$2.7\times 10^{-7}$ m s${}^{-1}$ and $k_{\mathrm{Sn}}=0.8$–$1.8\times 10^{-6}$ m s${}^{-1}$ in the temperature range of 1680∼1760 ${}^{{}^{\circ}}$C. Therefore, As and Sn evaporations are favorable at a high temperature. In view of the BOF, the later stage of the steelmaking would be favorable where the temperature of the steel is high. This is contrary to the finding in Section 4.1. Therefore, it is expected that during the oxygen top-blown steelmaking process, the C content and temperature work in the opposite way in accelerating the evaporation.

The activation energy of each evaporation reaction was evaluated by plotting the apparent rate constants in the Arrhenius form:

(5) $$\begin{array}{c} \ln k_{M}=-\frac{E_{A}^{M}}{RT}+C \end{array}$$

where $E_{A}^{M}$ is the activation energy of the evaporation of M from the droplet (J mol${}^{-1}$).

**Figure 4:**
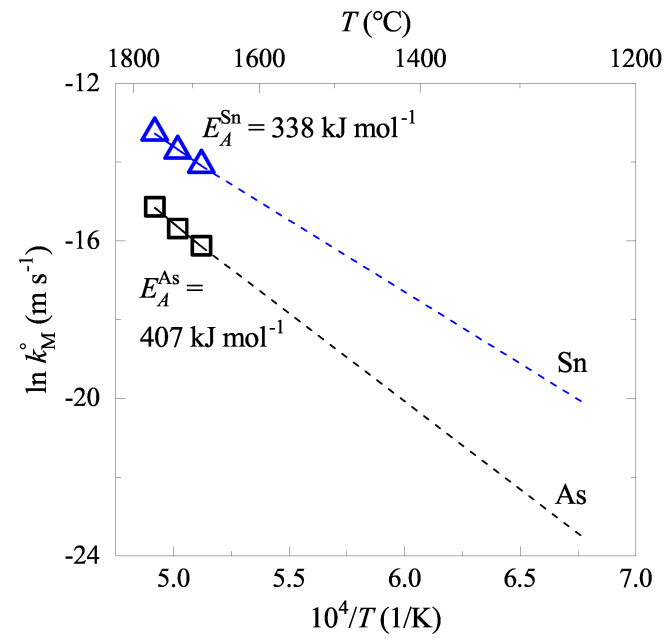
Arrhenius plot of the apparent rate constants of As and Sn. Dashed lines are the extrapolated to the lower temperature (near steelmaking temperature) from the temperature of the present experiment data.

Figure 4 shows the corresponding Arrhenius plots for As and Sn evaporations and the estimated $E_{A}^{M}$. The data show reasonable linearities, and $E_{A}^{\mathrm{As}}$ and $E_{A}^{\mathrm{Sn}}$ were obtained to be 407 kJ mol${}^{-1}$ and 338 kJ mol${}^{-1}$, respectively. Accordingly, $k_{\mathrm{As}}$ and $k_{\mathrm{Sn}}$ would be in the range of 10${}^{-10}$∼10${}^{-8}$ m s${}^{-1}$ and 10${}^{-9}$∼10${}^{-7}$ m s${}^{-1}$, respectively, in the temperature range of liquid iron (1250∼1650 ${}^{{}^{\circ}}$C) during the BOF process.

## 5. Evaporation of As and Sn from Liquid Iron during the Top-Blown Oxygen BOF Process

In the present study, a scenario of removal of the tramp elements (As and Sn) during the BOF process was envisaged. The following assumptions were made:

(1) Ferrous scrap was charged into hot metal at the beginning of the BOF process with a top-blown oxygen lance. The scrap melts and the tramp elements in the scrap dissolve in the hot metal.
(2) Once the oxygen blowing starts, some portion of hot metal splashes by forming small sizes of droplets. Each droplet stays out of the hot metal bath for a certain residence time (τ), which depends on the size and C content in the droplet. The temperature and C content in the droplets are different from those of the remaining hot metal bath. Those reported in the literature were used in the present study [21,22,23,27,28].
(3) As and Sn evaporate during the steelmaking process. These elements were considered to evaporate both from the liquid bath and the ejected droplets, where the latter were thought to contribute considerably to the evaporation kinetics of these elements, due to the large surface-to-volume ratio and high temperature.
(4) Two routes for the evaporation (liquid bath and droplets) for As and Sn take place independently, but the contents of these elements in the bath and the droplets are coupled with each other.
(5) The droplet surface is open to the gas phase, thereby allowing free evaporation of As and Sn without physical blocking by BOF slag. This is not unlikely as the bloated droplets are covered by decarburized gas [21,29]. On the other hand, the other surface blocking (chemically) by surface adsorption of O [30] was neglected, as its effect would appear at the last stage of the reaction.
(6) In order to avoid too complicated calculations due to the size variety, it was assumed that the droplet diameter is constant (2 mm), which falls in the middle of the reported values (1 to 2 mm [31], 0.23 to 3.35 mm [32]).

**Figure 5:**
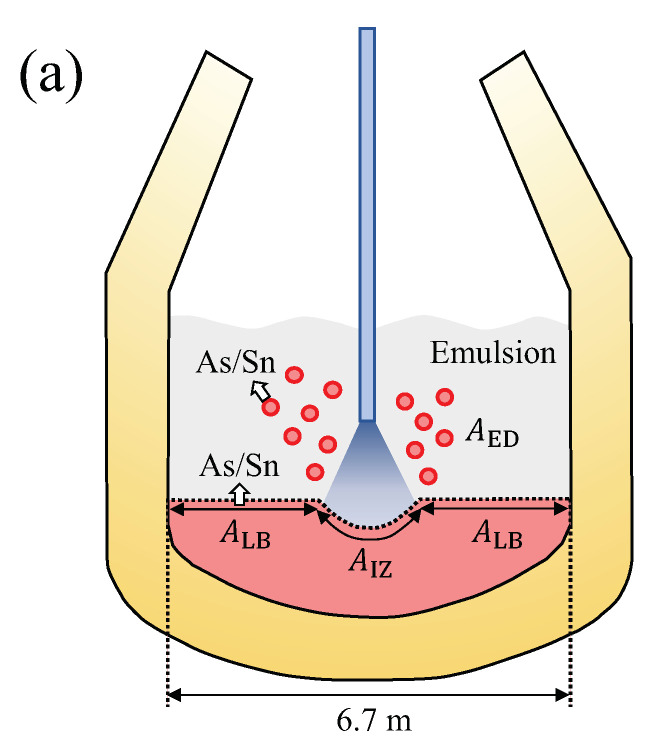

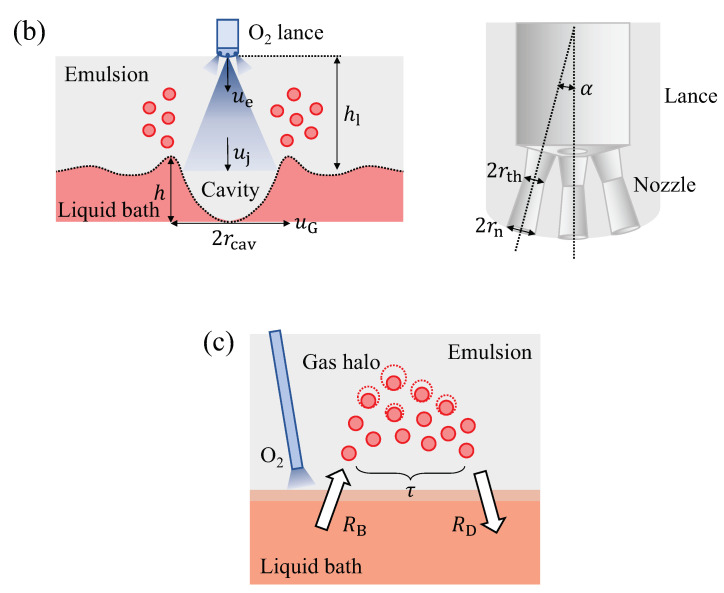
A schematic diagram of evaporation during the BOF process: (**a**) converter showing various places of evaporation (*D* = 6.7 m): $A_{\mathrm{ED}}$, $A_{\mathrm{IZ}}$, and $A_{\mathrm{LB}}$ are the surface areas of the ejected droplets, the impact zone forming the cavity, and rest of the surface of liquid bath, respectively; (**b**) nozzle configuration and cavity formation: *h*, $h_{l}$, $r_{\mathrm{cav}}$, $u_{e}$, $u_{j}$, and $u_{G}$ are the cavity height, the lance height, the radius of the cavity, the jet centerline velocity at nozzle exit, the jet centerline velocity at impact point, the critical tangential jet velocity, respectively; (**c**) droplet generation beneath the nozzle: $R_{B}$, $R_{D}$, and τ are the specific droplet generation rate, the specific droplet removal rate, and the residence time of the droplet, respectively.

**Figure 6:**
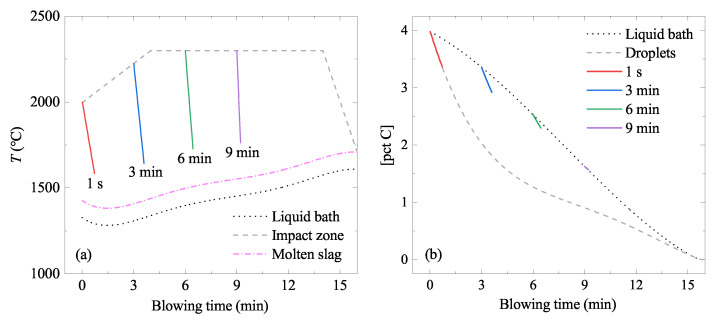
Change of (**a**) temperature and (**b**) [pct C] in various places in BOF. Solid curves were calculated in the present study for ejected droplets generated at 1 s, 3 min, 6 min, and 9 min, respectively, during the oxygen blowing. Dash-dot curve is the estimated temperature of the slag [28]. Dotted curves are those for the liquid bath [22]. The dashed curve in (**a**) is the temperature at the impact zone [23]. The dashed curve in (**b**) is the [pct C] in ejected droplets reported by Dering et al. [22]. (color online).

A schematic figure is seen in Figure 5 to describe the situation in the 200 tonne BOF. Temperature and C contents in various places in liquid iron are shown in Figure 6. Dashed curves and dotted curves were taken from the available literature [22,23,28]. Solid curves in the figures will be explained later (Section 5.1.5 and Section 5.1.6). All input parameters used for the calculation are listed in Table 2.

### 5.1. Ejected Droplets (ED) during the Oxygen Blowing

**Figure 7:**
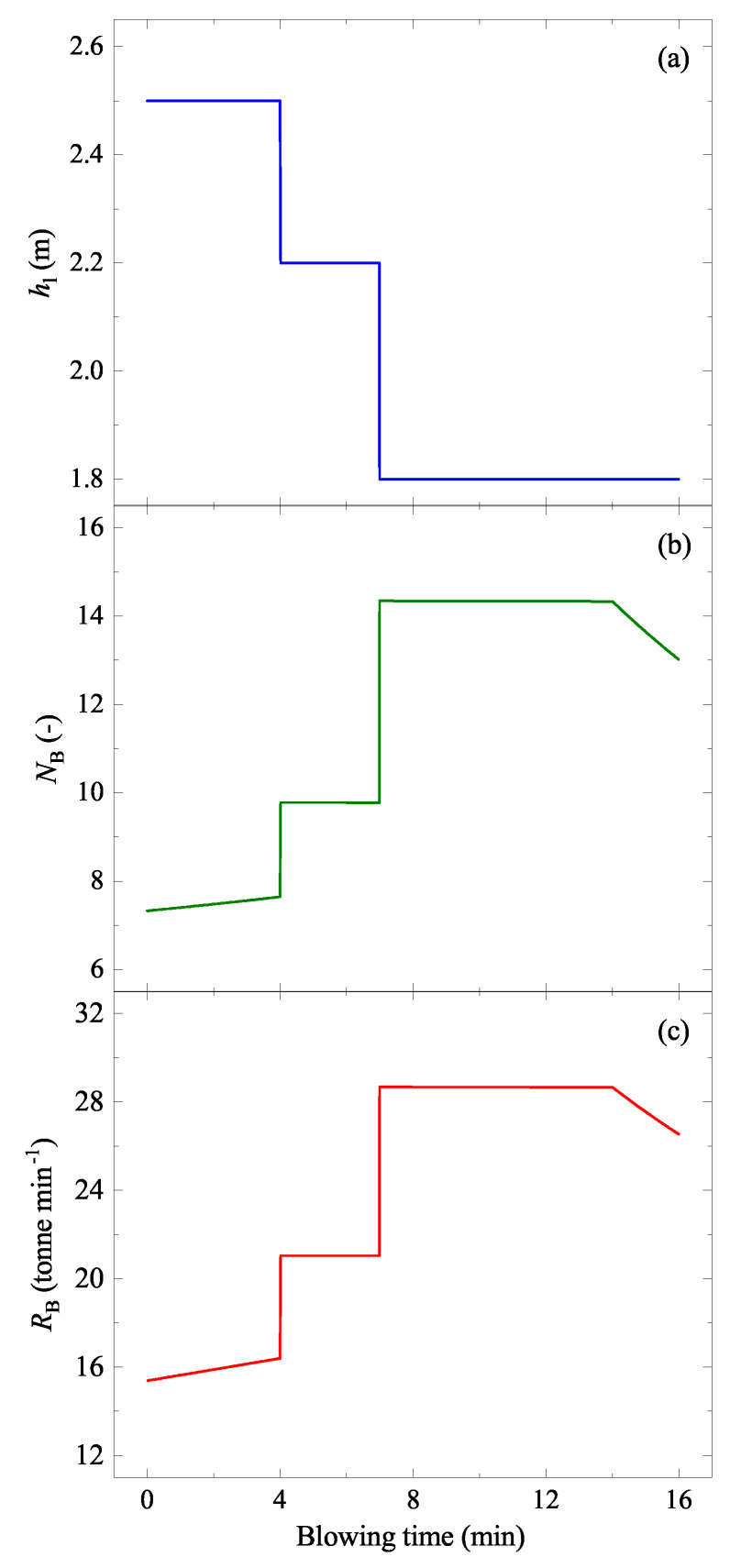
Assessed factors used in the model calculation: (**a**) lance height ($h_{l}$) [27,32], (**b**) blowing number ($N_{B}$), and (**c**) the droplet generation rate ($R_{B}$) as a function of the oxygen blowing time. (color online).

The change of the oxygen blowing lance height ($h_{l}$) is shown in Figure 7a [27,32]. In the study of Cicutti et al. [27,32], the flow rate ($F_{G}$, Nm${}^{3}$ min${}^{-1}$) was a constant (620 Nm${}^{3}$ min${}^{-1}$). By the oxygen blowing, several droplets were generated. Lin and Guthrie proposed that the droplets are generated at a specific droplet generation rate ($R_{B}$, kg min${}^{-1}$), stay for a certain residence time (τ, min) in the emulsion, and return to the bath at a specific droplet removal rate ($R_{D}$, kg min${}^{-1}$, see Figure 5c) [19]. Since the droplets are continuously generated during the oxygen blowing, [pct C] and the temperature in the droplets at each ejection moment are different.

#### 5.1.1. Droplet Generation Rate

The droplet generation rate ($R_{B}$) is highly dependent on the blowing conditions. Subagyo et al. investigated the behavior of droplets in the emulsion generated by impinging gas blowing [20]. Using their measured data and the low-temperature results of previous studies [36,37], they proposed an empirical correlation between the droplet generation rate per unit volume of blown gas and the blowing number ($N_{B}$) as follows:

(6) $$\frac{R_{B}}{F_{G}}=\frac{{\left(N_{B}\right)}^{3.2}}{{\left[2.6\times 10^{6}+2.0\times 10^{-4}{\left(N_{B}\right)}^{12}\right]}^{0.2}}$$

where $F_{G}$ is the flow rate of the gas (Nm${}^{3}$ min${}^{-1}$). $N_{B}$ was defined by Subagyo et al. [20] as:

(7) $$N_{B}=\frac{\rho_{G}u_{G}^{2}}{2\sqrt{\rho g\sigma }}$$

where $\rho_{G}$, ρ, $u_{G}$, *g*, and σ are the density of the gas (kg m${}^{-3}$), that of the liquid (kg m${}^{-3}$), the critical tangential jet velocity (m s${}^{-1}$), the gravitational acceleration (m s${}^{-2}$), and the surface tension of liquid (N m${}^{-1}$), respectively. The $u_{G}$ is related to the free turbulence jet axial velocity, $u_{j}$ [20,34,38,39]:

(8) $$u_{G}=0.44721\times u_{j}$$

where $u_{j}$ is the jet centerline velocity at impact point (m s${}^{-1}$). In the process of calculating $N_{B}$, $u_{j}$ can be expressed as a function of dimensional blowing conditions and the oxygen flow rate as follows [38]:

(9) $$\begin{array}{cc} u_{j} & =\frac{0.97u_{e}}{0.07\frac{h_{l}}{r_{n}}+0.29} \end{array}$$

(10) $$\begin{array}{cc} u_{e} & =\frac{F_{G}}{A_{n}} \end{array}$$

where $u_{e}$ is the jet centerline velocity at nozzle exit (m s${}^{-1}$), $r_{n}$ is the exit radius of nozzle (m), and $A_{n}$ is the nozzle cross-sectional area (= $\pi r_{n}^{2}$, m${}^{2}$), as shown schematically in Figure 5b. Equations (7)–(10) are substituted into Equation (6) in order to obtain $R_{B}$. In the present study, the reported geometry in the papers of Cicutti et al. [27,32] were used to calculated the $N_{B}$ and $R_{B}$. The results are shown in Figure 7b,c, respectively, along with the $h_{l}$ in Figure 7a. It is seen that the $N_{B}$ and $R_{B}$ are highly dependent on $h_{l}$.

#### 5.1.2. Residence Time of the Ejected Droplets (τ)

**Figure 8:**
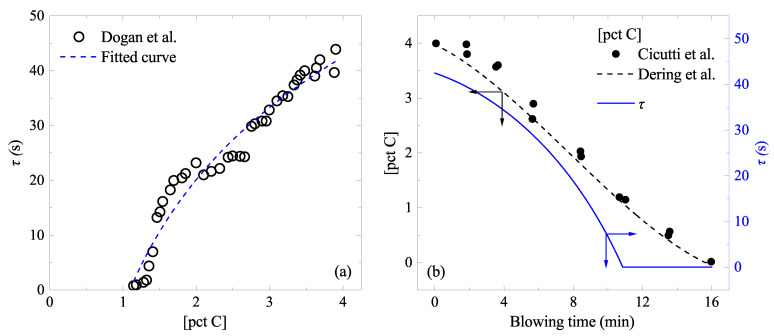
Residence time of the ejected droplets: (**a**) model predicted data (open circles) reported by Dogan et al. for the droplets having d=∼2 mm in the emulsion [21], as a function of [pct C]. (**b**) [pct C] change reported by Cicutti et al. (closed circles) [27,32]. Dashed curve was fitted to these two sets of data [22]. A solid curve is the estimated residence time as a function of the blowing time. It is to be noted that τ is seen to level-off [pct C]<∼1.1. This leads to an additional leveling-off in Figure 10, 11a, and 14. (color online).

τ (s) varies depending on [pct C] and the droplet size [21]. As was mentioned before, only a single-sized droplet was assumed in the present study for the sake of simplicity (*d* = 2 mm). In Figure 8a, τ of the droplets having approximately *d* = 2 mm was shown as a function of [pct C]. Open circles were taken from Dogan et al. [21]. Using the reported relationship between blowing time *t* (min) and [pct C] [22,27,32], as seen in Figure 8b (closed circles and a dashed curve), a relationship between τ and *t* was estimated:

(11) $$\tau (s)=-0.0137t^{3}-0.0605t^{2}-1.627t+42.660$$

which was also shown in Figure 8b by a solid curve. As seen in Figure 8a, the droplets do not eject at low [pct C] (approximately less than 1 pct). Therefore, the estimated τ was zero in the later stage of the blowing (t>11 min).

**Figure 9:**
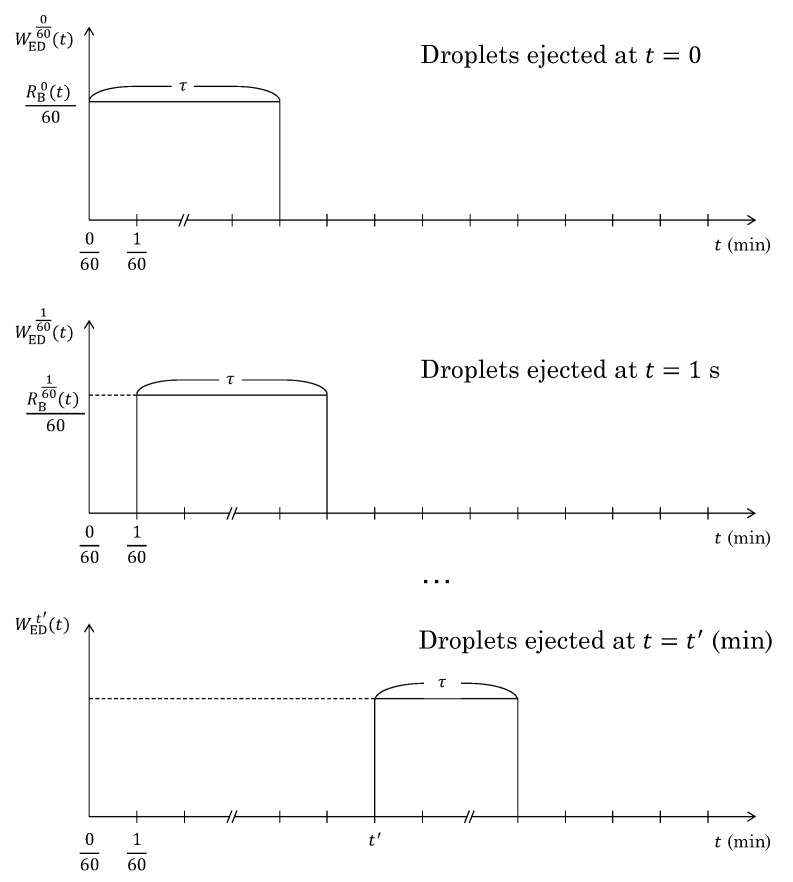
A schematic representation of the mass of the ejected droplets at each moment of the ejection ($t^{\prime }$) as functions of the blowing time *t* ($W_{\mathrm{ED}}^{t^{\prime }}\left(t\right)$). It should be noted that τ depends on [pct C] of the droplets at $t^{\prime }$.

**Figure 10:**
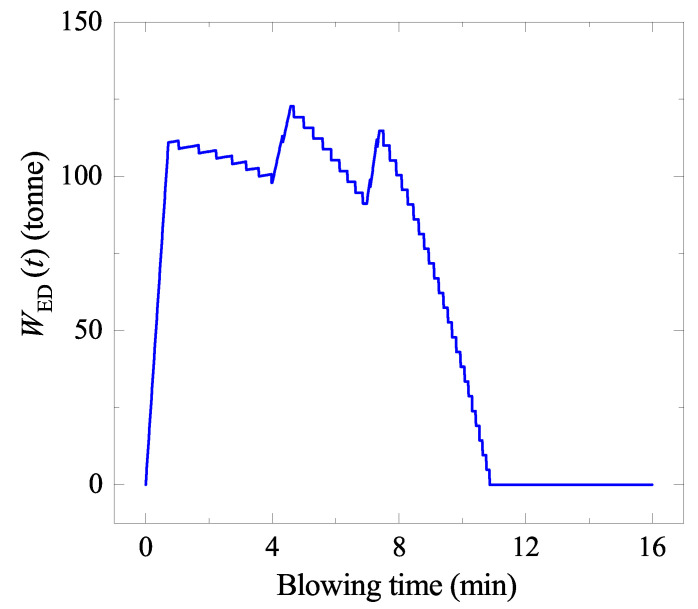
Mass of the ejected droplets staying in the emulsion ($W_{\mathrm{ED}}\left(t\right)$) as a function of blowing time.

**Figure 11:**
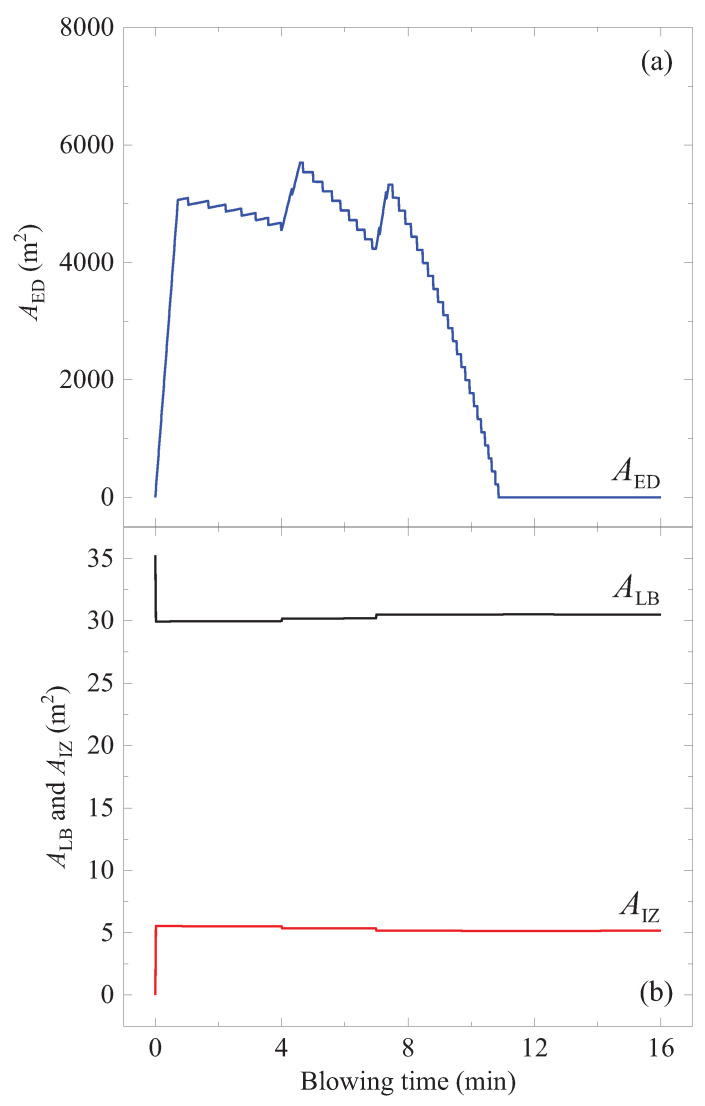
Area change during the oxygen blowing: (**a**) $A_{\mathrm{ED}}\left(t\right)$ for the ejected droplets, (**b**) $A_{\mathrm{IZ}}\left(t\right)$ for the impact zone in the liquid bath, and $A_{\mathrm{LB}}\left(t\right)$ for the rest of the liquid bath.

#### 5.1.3. Mass of the Ejected Droplets ($W_{\mathrm{ED}}$)

$W_{\mathrm{ED}}$, the mass of the droplets staying in the emulsion during the blowing, varies during the oxygen blowing. Therefore, it is indeed a function of the blowing time ($W_{\mathrm{ED}}\left(t\right)$). At each moment of the ejection ($t^{\prime }$), the mass of the ejected droplets ($W_{\mathrm{ED}}^{t^{\prime }}\left(t\right)$) would be approximated as shown in Figure 9 by $R_{B}^{t^{\prime }}\left(t\right)$ (the droplet generation rate at $t^{\prime }$), with a time interval Δt=1 s (1/60 min). This is based on the following relationship [19]:

(12) $$\frac{dW_{\mathrm{ED}}^{t^{\prime }}\left(t\right)}{dt}=R_{B}^{t^{\prime }}\left(t\right)-R_{D}^{t^{\prime }}\left(t\right)$$

where $R_{D}^{t^{\prime }}$ is zero while droplets stay in the emulsion, but is the same as $R_{B}^{t^{\prime }}$ once the droplets return to the bath as follows:

(13) $$R_{D}^{t^{\prime }}\left(t\right)=\left\{\begin{array}{cc} 0 & \left(t<t^{\prime }+\tau \right)\\ R_{B}^{t^{\prime }}\left(t\right) & \left(t^{\prime }+\tau \leq t\right) \end{array}\right.$$

It is thus formulated as:

(14) $$W_{\mathrm{ED}}^{t^{\prime }}\left(t\right)=\left\{\begin{array}{cc} 0 & \left(t<t^{\prime }\right)\\ \frac{R_{B}^{t^{\prime }}\left(t\right)}{60} & \left(t^{\prime }\leq t<t^{\prime }+\tau \right)\\ 0 & \left(t^{\prime }+\tau \leq t\right) \end{array}\right.$$

where the specific droplet generation rate was the function of *t*. Then, $W_{\mathrm{ED}}\left(t\right)$ is obtained as:

(15) $$W_{\mathrm{ED}}\left(t\right)=\sum_{t^{\prime }=0}^{t_{f}^{\prime }}W_{\mathrm{ED}}^{t^{\prime }}\left(t\right)$$

where $t_{f}^{\prime }$ is the last moment of the ejection. The estimated $W_{\mathrm{ED}}\left(t\right)$ along with the oxygen blowing condition (Figure 7) is shown in Figure 10.

#### 5.1.4. Area of the Ejected Droplets ($A_{\mathrm{ED}}$)

The surface area of an ejected droplet was simply assumed to be that of a sphere of the diameter *d*, and the number of droplets during the blowing was determined by dividing the total mass of droplets ($W_{\mathrm{ED}}\left(t\right)$) by the mass of one droplet. Therefore, the surface area of the droplets ejected at $t^{\prime }$ ($A_{\mathrm{ED}}^{t^{\prime }}\left(t\right)$) is obtained as:

(16) $$A_{\mathrm{ED}}^{t^{\prime }}\left(t\right)=\left(\pi d^{2}\right)\times \left(\frac{W_{\mathrm{ED}}^{t^{\prime }}\left(t\right)}{\frac{1}{6}\rho \pi d^{3}}\right)$$

where *d* was assumed to be 2 mm. Change of the density due to the change of C content and temperature was taken into account [26]. Then, $A_{\mathrm{ED}}\left(t\right)$ is obtained as:

(17) $$A_{\mathrm{ED}}\left(t\right)=\sum_{t^{\prime }=0}^{t_{f}^{\prime }}A_{\mathrm{ED}}^{t^{\prime }}\left(t\right)$$

In this way, the surface area change of all droplets during the blowing ($A_{\mathrm{ED}}$) was calculated, as shown in Figure 11a. The $A_{\mathrm{ED}}$ increases up to almost 163 times of the liquid bath surface area before the blowing.

#### 5.1.5. Temperature Change of the Ejected Droplets

It was assumed that the temperature of the droplet was the same as that of the impact zone (dashed curve in Figure 6a) at the moment of the ejection, but gradually decreased to a certain level. Madhavan et al. reported that the droplet ejected from the impact zone lost approximately 73 pct of the heat during its residence time (τ) due to the ambient slag [28]. The slag temperature was approximately 100 ${}^{{}^{\circ}}$C higher [28] than that of the liquid bath [22] (dotted curve in Figure 6a). This was simply interpreted as the temperature of each droplet decreasing from the temperature of the impact zone at the time of the ejection to a temperature that is 27 pct higher than that of the ambient slag, linearly in the residence time. Therefore, the temperature profile is different for each droplet, depending on their ejection time ($t^{\prime }$). Some calculated examples according to the above logic are shown in Figure 6a.

#### 5.1.6. [pct C] of the Ejected Droplets

Variations of [pct C] in the liquid bath and in the ejected droplets were reported by Dering et al. [22] (Figure 6b). It was assumed that the [pct C] in the droplet was the same as that of the liquid bath at the time of the ejection ($t^{\prime }$), but gradually decreased during the residence time (τ) following the [pct C] vs. *t* in the ejected droplet (dotted curve in Figure 6b). Some calculated examples according to the above logic are shown in Figure 6b.

### 5.2. Liquid Bath in the Converter

A converter with a diameter (*D*) of 6.7 m was considered [33]. After the start of the oxygen blowing, several cavities at impact zones can be formed on the surface of the bath, as seen in Figure 5b. The surface of a cavity was assumed to be paraboloid [40], and its area was calculated as a function of lance height and the oxygen flow rate:

(18) $$\begin{array}{c} A_{\mathrm{cav}}=\frac{\pi r_{\mathrm{cav}}^{4}}{6h^{2}}\left[\left(1+\frac{4h^{2}}{r_{\mathrm{cav}}^{2}}\right)-1\right] \end{array}$$

where $A_{\mathrm{cav}}$ is the surface area of the cavity (m${}^{2}$), *h* is the cavity height (m), and $r_{\mathrm{cav}}$ is the radius of the cavity (m) [41]. The *h* and $r_{\mathrm{cav}}$ can be expressed by using the dimensionless correlations proposed by Koria and Lange [42] as follows:

(19) $$\begin{array}{cc} h & =4.469h_{l}{\left(\frac{{\dot{m}}_{n}\cos\alpha }{\rho gh_{l}^{3}}\right)}^{0.660} \end{array}$$

(20) $$\begin{array}{cc} r_{\mathrm{cav}} & =1.407h_{l}{\left(\frac{{\dot{m}}_{n}\left(1+\sin\alpha \right)}{\rho gh_{l}^{3}}\right)}^{0.282} \end{array}$$

where α is the nozzle angle (${}^{{}^{\circ}}$) and ${\dot{m}}_{n}$ is the momentum flow rate of the each nozzle (kg m s${}^{-2}$). ${\dot{m}}_{n}$ is proportional to the total momentum flow rate (${\dot{m}}_{t}$, kg m s${}^{-2}$) and inversely proportional to the number of nozzles ($n_{n}$) by the following equations [42]:

(21) $$\begin{array}{cc} {\dot{m}}_{n} & =\frac{{\dot{m}}_{t}}{n_{n}} \end{array}$$

(22) $$\begin{array}{cc} {\dot{m}}_{t} & =0.7854\times {\left(2r_{\mathrm{th}}\right)}^{2}\times n_{n}\times P_{a}\left(\frac{1.27P_{0}}{P_{a}}-1\right) \end{array}$$

where $r_{\mathrm{th}}$ is the throat radius of the lance (m), $P_{0}$ and $P_{a}$ are the top supply pressure and the ambient pressure, respectively (Pa). When the α exceeds 10${}^{{}^{\circ}}$, the cavities formed by the nozzles do not overlap each other [41]. In the present study, the nozzle angle was set to 17.5${}^{{}^{\circ}}$ [21] and the area of impact zone ($A_{\mathrm{IZ}}$, m${}^{2}$) was estimated by multiplying the individual cavity area ($A_{\mathrm{cav}}$) by the number of nozzles in the lance tip:

(23) $$\begin{array}{c} A_{\mathrm{IZ}}=n_{n}A_{\mathrm{cav}} \end{array}$$

The input parameters used for the calculation were taken from literature [21,27,32,33,34] and are listed in Table 2. The lance height $h_{l}$ was taken from Cicutti et al. [27,32], as shown in Figure 7a. The calculated surface area of the impact zone ($A_{\mathrm{IZ}}$) during a blow is shown in Figure 11b. Surface areas of the liquid bath except for the impact zone ($A_{\mathrm{LB}}$) and of the ejected droplets ($A_{\mathrm{ED}}$) are also shown in the figure.

Mass of the liquid bath except for the ejected droplets is calculated as:

(24) $$W_{\mathrm{LB}}\left(t\right)=\mathrm{total}\;\mathrm{mass}\;\mathrm{of}\;\mathrm{liquid}\;\mathrm{iron}-W_{\mathrm{ED}}\left(t\right)$$

The total mass of liquid iron in the present study was set to 200 tonnes.

### 5.3. Rate Constant

The evaporation rate depends on the rate constant, area, volume, and content of each element (Equation (3)). These four terms are different at each place of the evaporation: the ejected droplet and the liquid bath. The ejected droplets during the whole oxygen blowing process were also distinguished by their ejected volume (proportional to $W_{\mathrm{ED}}^{t^{\prime }}\left(t\right)$), the area ($A_{\mathrm{ED}}^{t^{\prime }}\left(t\right)$), and the content of the evaporating species ([pct M]${}_{\mathrm{ED}}^{t^{\prime }}\left(t\right)$). $W_{\mathrm{ED}}^{t^{\prime }}\left(t\right)$ and $A_{\mathrm{ED}}^{t^{\prime }}\left(t\right)$ were already evaluated in Equations (14) and (16).

**Figure 12:**
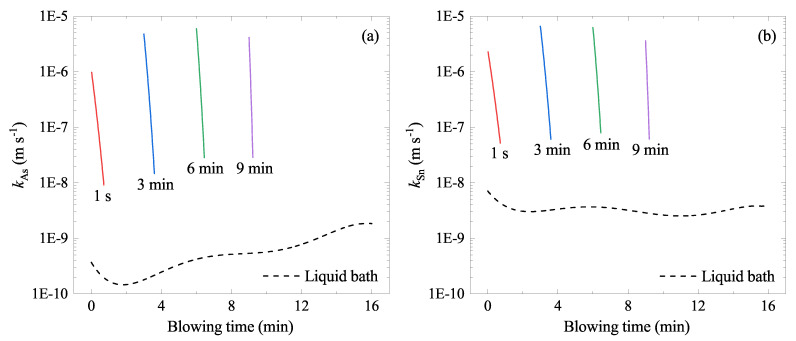
Change of the evaporation rate constant in the liquid bath and in the droplets ejected at $t^{\prime }$ = 1 s, 3 min, 6 min, and 9 min, respectively, during the oxygen blowing: (**a**) $k_{\mathrm{As}}$ (**b**) $k_{\mathrm{Sn}}$. Solid curves are those for the ejected droplets, and dashed curves are those for the liquid bath.

The rate constant was modeled as follows. The experimentally measured rate constant in Figure 4 were fitted to the Arrhenius equation as:

(25) $$\begin{array}{ccc} \ln k_{\mathrm{As}}^{{}^{\circ}} & = & \frac{-48,990}{T}+8.9370 \end{array}$$

(26) $$\begin{array}{ccc} \ln k_{\mathrm{Sn}}^{{}^{\circ}} & = & \frac{-40,662}{T}+6.7251 \end{array}$$

which were measured at C saturation (*T* in K). Jung et al. reported that the evaporation rate constant of Cu from liquid Fe–C alloys were proportional to the activity coefficient of Cu ($f_{\mathrm{Cu}}$), which could be described by the Wagner’s interaction parameter formalism [9]. Therefore, the rate constants ($k_{\mathrm{As}}$ and $k_{\mathrm{Sn}}$) at low [pct C] were estimated as follows:

(27) $$k_{M}=k_{M}^{{}^{\circ}}\left(\frac{f_{M}}{f_{M}^{{}^{\circ}}}\right)$$

where ${}^{{}^{\circ}}$ represents the C saturation condition. The activity coefficient of M is:

(28) $$\log f_{M}=e_{M}^{C}[\mathrm{pct}\;C]$$

where $e_{M}^{C}$ is the first-order interaction parameter between M and C when mass pct is used as the content variable. $e_{M}^{M}[\mathrm{pct}\;M]$ was neglected due to low [pct M]. $e_{\mathrm{As}}^{C}=0.25$, $e_{\mathrm{Sn}}^{C}=0.37$ at 1600 ${}^{{}^{\circ}}$C were taken from Lupis [43]. Their temperature dependence was estimated assuming the regular solution behavior:

(29) $$\epsilon_{M}^{C}\left(T\right)=\frac{1873}{T}\times \epsilon_{M}^{C}\left(\mathrm{at}\;1600\;{}^{{}^{\circ}}C\right)$$

(30) $$e_{M}^{C}\left(T\right)=\frac{M_{\mathrm{Fe}}}{230M_{C}}\times \left[\epsilon_{M}^{C}\left(T\right)-\frac{M_{\mathrm{Fe}}-M_{C}}{M_{\mathrm{Fe}}}\right]$$

where $\epsilon_{M}^{C}\left(T\right)$ is the first-order interaction parameter between M and C at *T* when mole fraction is used as the content variable, $M_{i}$ is the atomic mass of the element *i*. Using the $e_{M}^{C}$ at 1600 ${}^{{}^{\circ}}$C and the experimentally determined $k_{M}$ (Equations (25) and ()), the $k_{M}$ at various temperature and [pct C] were estimated for the liquid bath and the ejected droplets. Since temperature and [pct C] in the liquid bath and in the ejected droplets vary during the blowing time, the $k_{M}$s also vary during the reaction. The calculated $k_{\mathrm{As}}$ and $k_{\mathrm{Sn}}$ in the liquid bath are shown by dashed curves in Figure 12. In addition to this, those of droplets ejected at different times ($t^{\prime }$) were also plotted by solid curves. In general, $k_{\mathrm{Sn}}$ was higher than $k_{\mathrm{As}}$, therefore, Sn would evaporate faster than As. $k_{\mathrm{Sn}}$ and $k_{\mathrm{As}}$ in the liquid bath vary in the order of $10^{-9}$ to $10^{-8}$ m s${}^{-1}$ and $10^{-10}$ to $10^{-9}$ m s${}^{-1}$, respectively. Those in the ejected droplets were considerably higher—varying in the order of $10^{-8}$ to $10^{-5}$ m s${}^{-1}$. Therefore, it was postulated that the higher temperature of the ejected droplet would give a higher evaporation rate. Moreover, significantly enlarged surface area ($A_{\mathrm{ED}}^{t^{\prime }}\left(t\right)$) can be coupled to increase the overall evaporation rate of As and Sn.

### 5.4. Evaporation Rate of As and Sn

The evaporation rate was assumed to follow the first-order reaction (3), both applied to the liquid bath and the ejected droplets. The content of M in the liquid bath at time *t* was designated by [pct M]${}_{\mathrm{LB}}\left(t\right)$, which represents an *average* content in the liquid bath. Similarly, the content of M in the droplets at time *t*, which had ejected at time $t^{\prime }$, was designated by [pct M]${}_{\mathrm{ED}}^{t^{\prime }}\left(t\right)$. Both are calculated as follows:

(31) $$\begin{array}{ccc} {}[\mathrm{pct}\;{M]}_{\mathrm{LB}}\left(t+\Delta t\right) & = & [\mathrm{pct}\;{M]}_{\mathrm{LB}}\left(t\right)\times \exp\left(-\frac{\left(A_{\mathrm{LB}}\left(t\right)+A_{\mathrm{IZ}}\left(t\right)\right)\rho k_{M}}{W_{\mathrm{LB}}\left(t\right)}\Delta t\right) \end{array}$$

(32) $$\begin{array}{ccc} {}[\mathrm{pct}\;{M]}_{\mathrm{ED}}^{t^{\prime }}\left(t+\Delta t\right) & = & [\mathrm{pct}\;{M]}_{\mathrm{ED}}^{t^{\prime }}\left(t\right)\times \exp\left(-\frac{A_{\mathrm{ED}}^{t^{\prime }}\left(t\right)\rho k_{M}}{W_{\mathrm{ED}}^{t^{\prime }}\left(t\right)}\Delta t\right) \end{array}$$

Δt was set to 1 s. The average content of M in the whole liquid iron was then obtained as:

(33) $$[\mathrm{pct}\;M]\left(t\right)=\frac{[\mathrm{pct}\;{M]}_{\mathrm{LB}}\left(t\right)W_{\mathrm{LB}}\left(t\right)+\sum_{t^{\prime }=0}^{t_{f}}[\mathrm{pct}\;{M]}_{\mathrm{ED}}^{t^{\prime }}\left(t\right)W_{\mathrm{ED}}^{t^{\prime }}\left(t\right)}{W_{\mathrm{LB}}\left(t\right)+W_{\mathrm{ED}}\left(t\right)}$$

**Figure 13:**
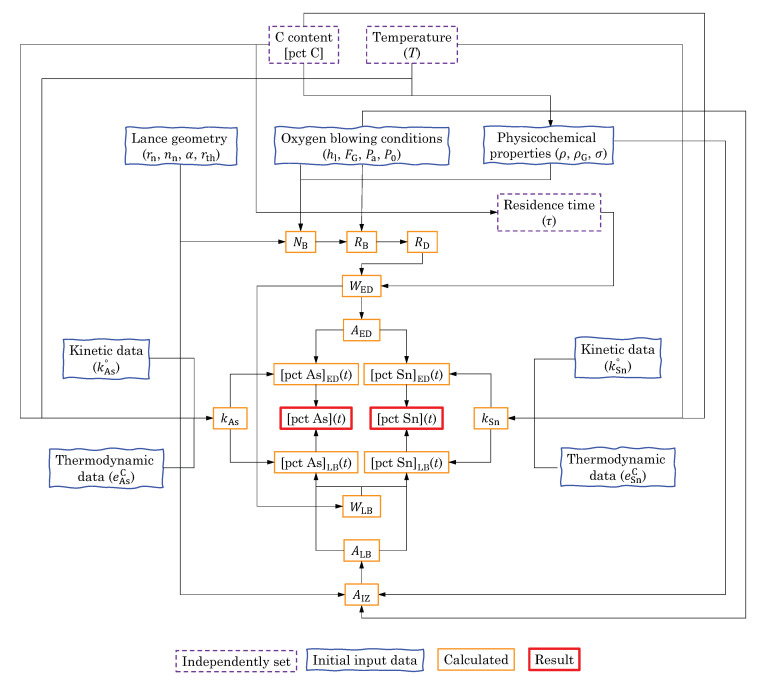
A flowchart of the steps of the present kinetic model calculation. (color online).

**Figure 14:**
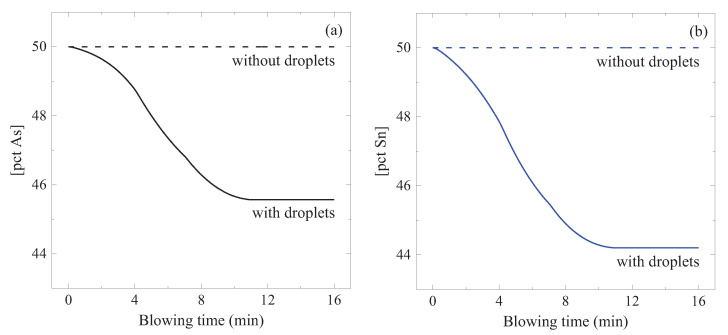
Model simulation results for the evaporation of (**a**) As and (**b**) Sn using the present model. Dashed and full lines are the evaporation results without and with the consideration of the ejected droplets, respectively. (color online).

A flow chart of the model calculation is shown in Figure 13. This model was used to simulate the change of [pct As] and [pct Sn] in a 200-tonne converter during the oxygen blowing. Initial contents were set to 50 ppm for both elements. The oxygen blowing condition (Figure 7), temperature, and [pct C] changes (Figure 6) were considered. The calculated results are shown in Figure 14. [pct As] and [pct Sn] decreased gradually with the oxygen blowing, but they did not decrease any more approximately after 10 min. Final [pct As] and [pct Sn] were 46 ppm and 44 ppm, respectively. As seen in the figure, most of the As and Sn evaporated from the ejected droplets. This can be attributed to the following considerations:

1. Wider reaction area $A_{\mathrm{ED}}$ than $A_{\mathrm{LB}}$ (Figure 11).
2. Higher rate constant ($k_{\mathrm{As}}$ and $k_{\mathrm{Sn}}$) in the ejected droplets than that in the liquid bath (Figure 12), due to the higher local temperature of the droplets (Figure 6).

Shown by dashed lines and dotted lines in Figure 14 are the model calculation results assuming that (1) the evaporation only takes place at the bath (evaporation at the droplets is ignored), and (2) the evaporation takes place at both places (bath and droplets) but the temperature at the droplets was the same as that in the bath, respectively. Both calculation results show that the evaporation rates of As and Sn were significantly slowed down, and the above two facts were indeed meaningful.

Nevertheless, the evaporation of As and Sn, even with the considerations of the above two, is still too slow to be practically meaningful. This implies that the evaporative refining of As and Sn should be carried out under coupled favorable conditions including high S in the case of Sn [6,7,8,12,13,14,15]. Vacuum degassing should be beneficial [16]. This requires a new process for the tramp element removal from the obsolete ferrous scraps.

## 6. Discussions

The liquid iron in the BOF system was considered in two parts: liquid bath and ejected iron droplets by oxygen blowing. Since the evaporation kinetics of these elements significantly depend on C content, temperature, and surface area exposed to gas, models or formulae describing these in the liquid bath and in the ejected iron droplets are necessary. In the present study, the available knowledge in the literature was manipulated to formulate the C contents and temperature of the liquid bath and those of the ejected droplets. The surface area of the liquid bath was estimated by the model of Cheslak et al. [40], considering cavity formation in the bath. The surface area of the ejected iron droplets was estimated using the residence time and the droplet generation rate obtained by Dogan et al. [21] and Subagyo [20], respectively. The evaporation rate constant was modeled to be dependent on C content and temperature, where the present experimental results for the evaporation rate constant at C saturation conditions were used. By coupling the C content, temperature, the evaporation rate constant, and the surface area of both liquid bath and ejected droplets, the evaporation rates of As and Sn were predicted. In a model system of a 200-tonnes BOF with a given oxygen blowing condition (Figure 7), As content decreased from 50 ppm to 46 ppm and Sn content decreased from 50 ppm to 44 ppm. These predictions were based on utilizing high temperature and wide surface area of the ejected droplets.

One of the limitations of the present approach is the assumption of the free surface on the ejected droplets. Some parts of the surface would be covered by BOF slag, which was neglected in the present study. Although self-evaporation of As and Sn seems less likely, the gas-halo formed by CO evolution by the bloated-droplet theory should provide some room for the As/Sn evaporation [21,29].

## 7. Conclusions

In the present study, the feasibility of As and Sn removal in liquid iron during the BOF process was assessed. A high-temperature electromagnetic levitation technique was employed to measure intrinsic kinetic parameters (evaporation rate constant of As and Sn). An evaporation rate model was subsequently developed in order to predict concentration changes of As and Sn during the top-blowing BOF process. The following conclusions were obtained:

1. Evaporation rates of As and Sn from liquid iron were slow: a small crucible test showed that As and Sn hardly evaporated at 1400 ${}^{{}^{\circ}}$C.
2. The electromagnetic levitation test showed that the evaporation rates increased by increasing temperature and C content in the liquid iron. The evaporation rate constants ($k_{\mathrm{As}}^{{}^{\circ}}$, $k_{\mathrm{Sn}}^{{}^{\circ}}$) were formulated as functions of temperature and C content using available thermodynamic data.
3. Mass, volume, and surface area of liquid iron in a 200-tonne BOF during the top-blowing were modeled using available information in the literature. The surface area of the liquid increased enormously (up to ∼163 times). This increased the site for evaporation, thereby increasing the evaporation rates.
4. Temperature of liquid steel at the impact zone was significantly high [23]. This resulted in high temperature in the ejected droplets, thereby increasing the evaporation rates.
5. Considering the enlarged surface area and the high temperature of many numbers of ejected droplets could enhance the evaporation. However, due to the too-low vapor pressure of As and Sn, resultant evaporation rates were not acceptable in practical operation. This suggests additional actions, including increasing vapor pressure of the tramp elements, e.g., adding S and decreasing ambient pressure, etc.

## Figures and Tables

**Table 1:** Initial composition of the samples ([pct M]${}_{0}$) used in the present study.

Case	Temperature (${}^{{}^{\circ}}$C)	[pct C]${}_{0}$	[pct As]${}_{0}$	[pct Sn]${}_{0}$	Crucible
1-1	1680 (±10)	2.0	0.18	0.18	Magnesia
1-2		3.9			
1-3		5.6 (sat.)	0.16	0.19	Graphite
2-1					
2-2	1720 (±10)	5.8 (sat.)			
2-3	1760 (±10)	5.9 (sat.)			

**Table 2:** Input parameters used for the model calculations. *T* in ${}^{{}^{\circ}}$C.

Input Parameters	Unit	Value
Diameter of converter	m	6.7 [33]
Lance height, $h_{l}$	m	1.8–2.5 (Figure 7) [27,32]
Nozzle angle, α	deg(${}^{{}^{\circ}}$)	17.5 [21]
Throat radius of nozzle, $r_{\mathrm{th}}$	m	0.017 [21]
Exit radius of nozzle, $r_{n}$	m	0.023 [21]
Number of nozzles, $n_{n}$	-	6 [27,32]
Supply pressure, $P_{0}$	Pa	10${}^{6}$ [21]
Ambient pressure, $P_{a}$	Pa	10${}^{5}$ [34]
Oxygen flow rate, $F_{G}$	Nm${}^{3}$ min${}^{-1}$	620 [27,32]
Mass of iron	tonne	200 [27,32,33]
Density of iron, ρ	kg m${}^{-3}$	7100 − 73.2 [pct C] − (0.828 − 0.0874 [pct C])
		× (*T* − 1550) [26]
Surface tension of iron, σ	N m${}^{-1}$	[(2367 ± 500) − 0.34 *T* ] / 1000 [35]
Density of oxygen, $\rho_{G}$	kg min${}^{-3}$	1.429 at STP
